# Metallographic Mechanism of Embrittlement of 15 μm Ultrafine Quaternary Silver Alloy Bonding Wire in Chloride Ions Environment

**DOI:** 10.3390/ma16031066

**Published:** 2023-01-25

**Authors:** Jun-Ren Zhao, Fei-Yi Hung, Che-Wei Hsu

**Affiliations:** Department of Materials Science and Engineering, National Cheng Kung University, Tainan 701, Taiwan

**Keywords:** silver wire, wire bonding, chloride ions, ion migration, galvanic corrosion

## Abstract

Chloride ions contained in the sealing compound currently used in the electronic packaging industry not only interact with intermetallic compounds but also have a serious impact on silver alloy wires. A 15 μm ultrafine quaternary silver-palladium-gold-platinum alloy wire was used in this study. The wire and its bonding were immersed in a 60 °C saturated sodium chloride solution (chlorination experiment), and the strength and elongation before and after chlorination were measured. Finally, the fracture surface and cross-section characteristics were observed using a scanning electron microscope and focused ion microscope. The results revealed that chloride ions invade the wire along the grain boundary, and chlorides have been generated inside the cracks to weaken the strength and elongation of the wire. In addition, chloride ions invade the interface of the wire bonding to erode the aluminum substrate after immersing it for enough long time, causing galvanic corrosion, which in turn causes the bonding joint to separate from the aluminum substrate.

## 1. Introduction

Wire bonding is the most widely used technology in the electronic packaging industry [1,2,3]. Previously, gold wire was the most commonly used material in wire bonding, but with the high price of gold, many alternative materials have been proposed [4,5,6]. Currently, the alternative materials with the most potential are copper-based and silver-based wires [7,8,9,10]. The most attention has been paid to palladium-coated copper wire [11,12]. Pure copper wires easily oxidize and corrode, causing reliability concerns. This shortcoming has been overcome by modifying the surface of the palladium coating. Silver-based wires are mainly used in the form of silver alloy [13,14] and gold-coated silver wires [15,16]. According to research [11,17,18], adding palladium to silver-based wires results in a palladium-rich single-phase layer at the bonding interface, which can inhibit the formation of intermetallic compounds (IMCs). In addition, adding gold and palladium simultaneously helps improve the electromigration effect and increases the life cycle of silver-based wires.

This study used the newly developed quaternary silver-palladium-gold-platinum alloy (coded APAP) wire. The solid solution of the platinum element can further improve the efficiency of wire drawing [19,20], resulting in a wire diameter as low as 15 μm. This is the finest wire diameter in the microelectronic packaging industry at present [21]. A fine wire diameter has the advantages of a higher pin count and lower feeding costs. This is a new-generation material with great potential.

Currently, the sealant used in the microelectronic packaging industry contains chloride ions [22,23]; thus, the wire is prone to reliability problems in relation to electrical and mechanical properties and disconnection. Studies [24,25] have also noted that after the copper-based wire and aluminum substrate have been welded, IMCs are susceptible to corrosion caused by chloride ions and decomposition, leading to bonding peeling, which greatly reduces reliability. Few studies have investigated the effect of chloride ions on alloy wires [1,23], and investigations into the influence of chloride ions on a silver-based wire are lacking.

The chlorination of silver-based wires has rarely been studied, and the development of related test methods and mechanisms remains insufficient. Therefore, a newly developed quaternary Ag-2.5 Pd-1.5 Au-0.15 Pt (wt.%) alloy wire was used in this study, as well as an independently developed chlorination test method to analyze and clarify the chlorination mechanism. The results can be used as a reference for the microelectronic packaging industry.

## 2. Experimental Procedures

A 15 μm diameter APAP wire from the wire drawing process was the research material in this study (general silver wire diameter of 20 μm). After the wire was soaked in a saturated sodium chloride solution (concentration of chloride ions: 16.5 wt.%) at 60 °C for 4 h, it was washed with deionized water and dried. The wire surfaces were observed under a scanning electron microscope (SEM; HITACHI SU-5000, HITACHI, Tokyo, Japan). A micro-tensile tester was used to investigate the changes in the tensile mechanical properties of the silver alloy wire during immersion in the saturated sodium chloride solution at different times. The initial strain rate and gauge length were 5 × 10^−3^ s^−1^ and 50 mm, respectively. The tensile data were the average of five tests.

The tensile fracture and cross-section surface were observed, and the profile was analyzed using a dual-beam focus ion beam (FIB; FEI Nova 200, FEI, Hillsboro, OR, USA), and energy dispersive spectroscopy (EDS) was employed to analyze the inside of the cracks to clarify the chlorine embrittlement fracture mechanism of the wire. Finally, a chlorination experiment was conducted on the first and second bonds of the APAP wire on the aluminum substrate (the downforce of the first and second bonds was 20 and 60 gf, respectively). The chlorination holding time was 10 min and 30 min, and the temperature was 60 ℃. Subsequently, the chlorination condition of the fracture and the bonding positions were observed through the micro-tensile test, FIB, and EDS element analysis.

## 3. Results and Discussion

### 3.1. Fracture Effect of Chloride Ions: APAP Wire

**Figure 1:**
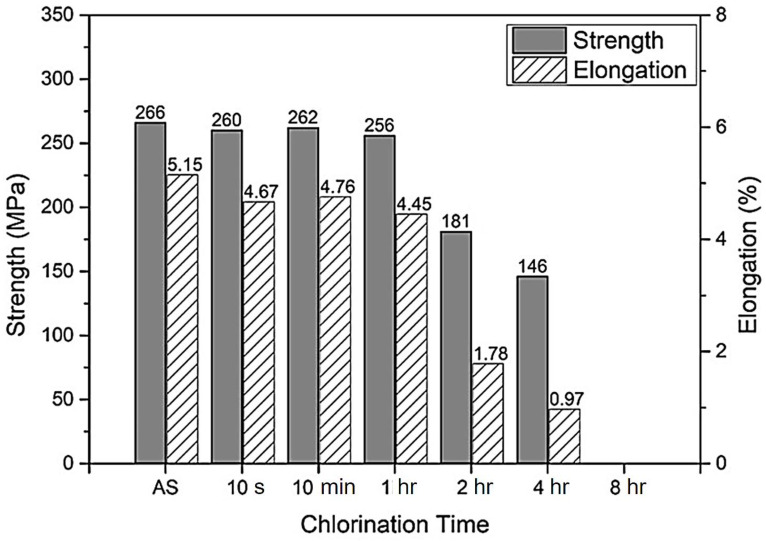
Changes in tensile strength and elongation of the APAP wires after immersion in saturated sodium chloride solution. (The tensile data were the average of five tests).

Figure 1 presents the change in the tensile strength (UTS) and elongation of the APAP wires immersed in the saturated sodium chloride solution for 10 s, 10 min, 1 h, 2 h, 4 h, and 8 h. According to the results, no significant difference in the strength and elongation of the wire within 1 h of immersion was detected, but as the immersion time increased to more than 2 h, a significant decrease in strength and elongation was observed, indicating that the corrosion caused by chloride ions has a considerable influence on the reliability of the wire. After being immersed for 8 h, the wire broke when tensile, indicating that the APAP wire cannot be used in a chlorinated environment for long periods.

**Figure 2:**
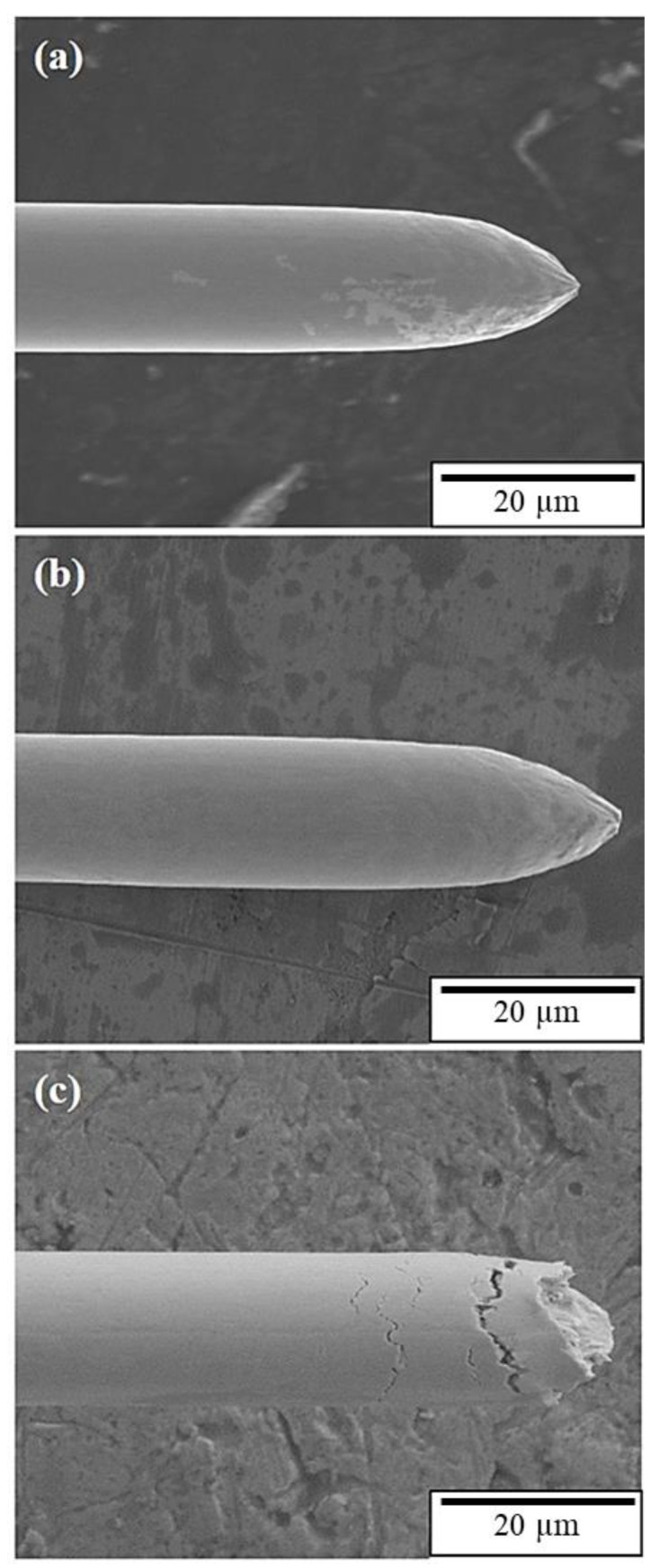
Tensile fracture surface of the APAP wire at different chlorination times: (**a**) before chlorination, (**b**) 2 h, and (**c**) 4 h.

**Figure 3:**
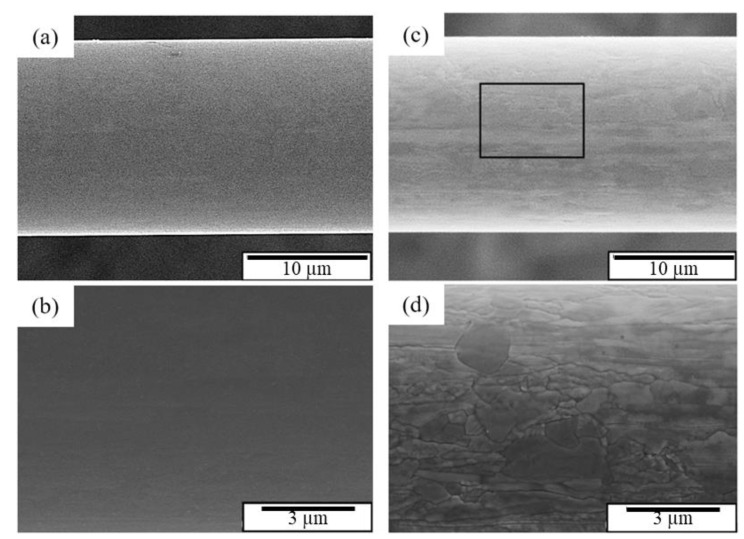
Surface morphology of the APAP wire: (**a**) macroscopic image before chlorination, (**b**) local magnified image before chlorination, (**c**) macroscopic image after chlorination for 4 h, and (**d**) local magnified image after chlorination for 4 h.

The tensile fracture surface of the APAP wire before and after chlorination was observed using an SEM (Figure 2). According to the literature [1], the fracture morphology of general metal wires is conical (the fracture mode is dominated by ductility). The APAP wire exhibited ductile failure characteristics before chlorination and after chlorination for 2 h. After chlorination for 4 h, the failure characteristics changed from ductile to brittle. After immersion in the saturated sodium chloride solution for 4 h, numerous fine cracks were distributed on the surface of the wire (Figure 3b,c), which was different from the wire before immersion in the saturated sodium chloride solution (Figure 3a,b). When the wire was subjected to tensile stress, these small cracks gradually connected to form large cracks, which eventually led to brittle fractures.

**Figure 4:**
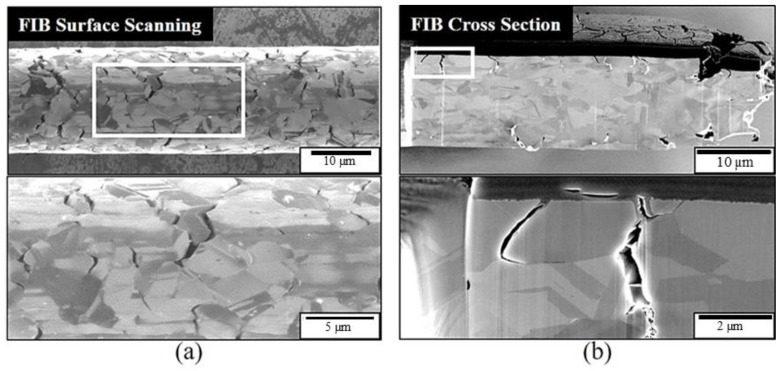
Tensile fracture cross-section of the APAP wire after 4 h of chlorination: (**a**) focus ion beam (FIB) surface scanning, and (**b**) cross-section image.

**Figure 5:**
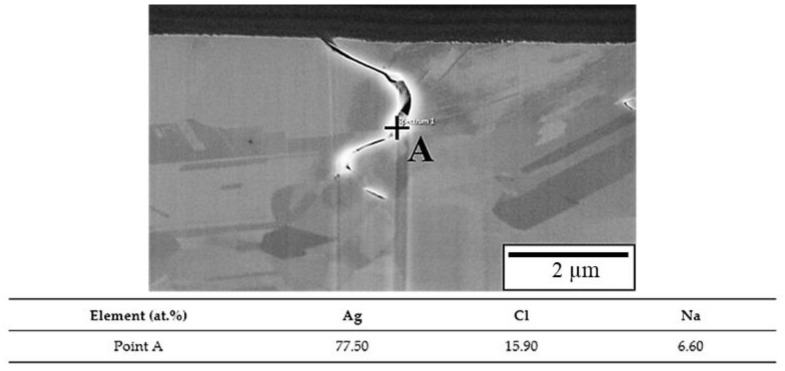
Energy dispersive spectroscopy (EDS) element analysis results of the crack.

To clarify the chlorination mechanism of the APAP wire, FIB scanning and profile analysis were performed on the fracture surface after chlorination for 4 h (Figure 4). Figure 4a presents the surface features 100 μM from the fractured surface. In addition to a large number of cracks on the surface of the wire, most cracks are located at the boundary of different color blocks. This indicates that the location of the cracks is at the grain boundary. Figure 4b presents the FIB profile analysis, and the microstructure was similar to our previous coated silver wire study [26]. The results demonstrate that the cracks penetrate deep into the wire along the grain boundary, and the longest crack is approximately 5 μm. Figure 5 presents the EDS element analysis results inside the cracks, revealing that chlorides have been generated inside the cracks. This demonstrates that chloride ions penetrate the wire through the grain boundary to form chlorides, causing the grain boundary to weaken.

**Figure 6:**
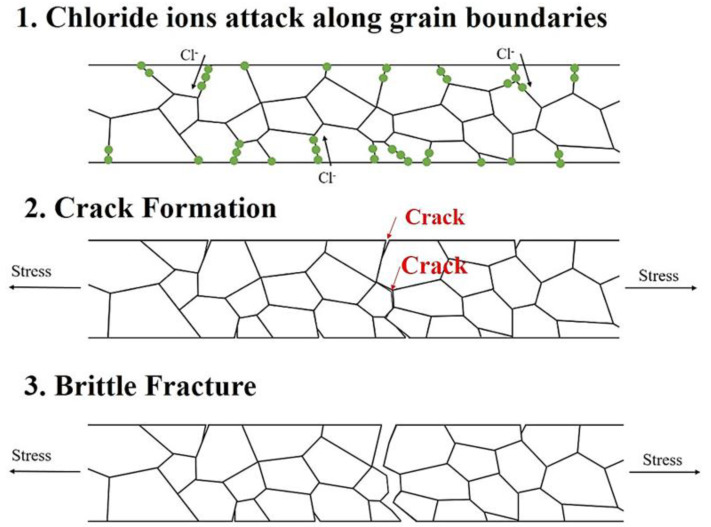
Intergranular brittleness fracture mechanism.

During the tensile test, the core not affected by chloride ions remained connected after the surface was broken and continued to deform. The cracks gradually widened from the inside to the outside, gradually penetrating the wire because of stress concentration. Finally, a brittle intergranular fracture occurred. The brittle fracture mechanism of the silver alloy wire is illustrated in Figure 6. The chlorine embrittlement phenomenon that occurs in silver alloy wires is caused by foreign chlorine ions that gradually erode the inside of the wire along the grain boundary or defects. Subsequently, a brittle fracture occurs when the wire is subjected to strain. The weakening of the grain boundary causes decreases in the tensile strength and elongation.

As the wire diameter gradually shrank, subtler phenomena, such as electromigration and chloride ion diffusion, became more evident. Another study [27] revealed that the silver-based wire exhibits a weakened grain boundary and intergranular fracture caused by electromigration during the electrification process. This is because the wires used today are mostly narrower than 20 μm in diameter; thus, even small changes cause major changes to the wire. For example, the longest crack in this study was 5 μm, and the unaffected wire diameter at the center was only 5 μm, too. This highlights the influence of the chlorine embrittlement phenomenon.

### 3.2. Fracture Effect of Chloride Ions: APAP Wire Bondingm

**Figure 7:**
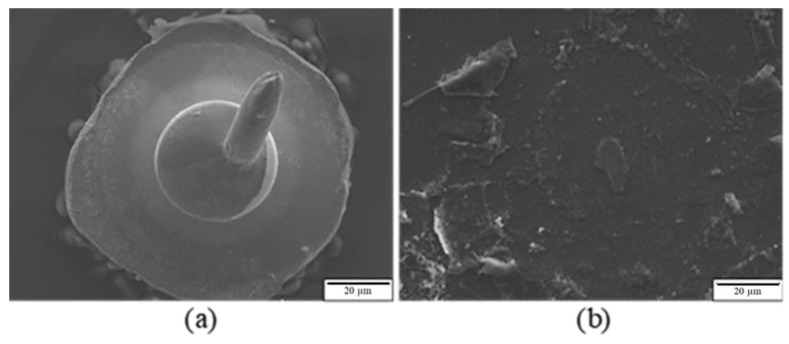
Fracture position of the first bond of the APAP wire after chlorination: (**a**) 10 min and (**b**) 30 min.

**Figure 8:**
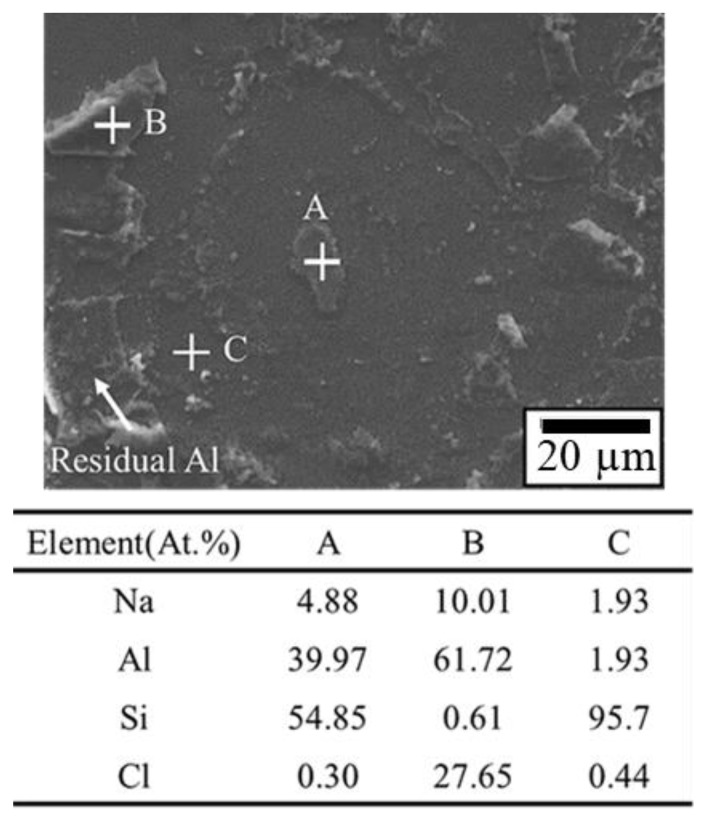
EDS analysis of the fracture surface of the first bond of the APAP wire after 30 min of chlorination. (A, B: residual aluminum film; C: silicon substrate).

Figure 7 presents the tensile fracture position of the first bond of the APAP wire immersed in a saturated sodium chloride solution at 60 °C for 10 and 30 min. After 10 min of chlorination, the first bond fracture position occurred in the heat-affected zone (Figure 7a), indicating that it retained some strength under these conditions. However, as the chlorination time increased to 30 min, the first bond joint separated from the aluminum substrate, and some traces remained on the substrate (Figure 7b). This suggests that after a long period of chlorination, chloride ions corrode the joint surface of the first bond, possibly causing the bond joint to separate and reducing reliability. Figure 8 presents the EDS analysis results of the fracture surface after 30 min of chlorination of the first bond joint of the APAP wire. The aluminum film on the substrate has been eroded by chloride ions and has peeled off. Points A and B in the figure denote the residual aluminum film, and point C is the exposed silicon substrate after the aluminum film has peeled off.

**Figure 9:**
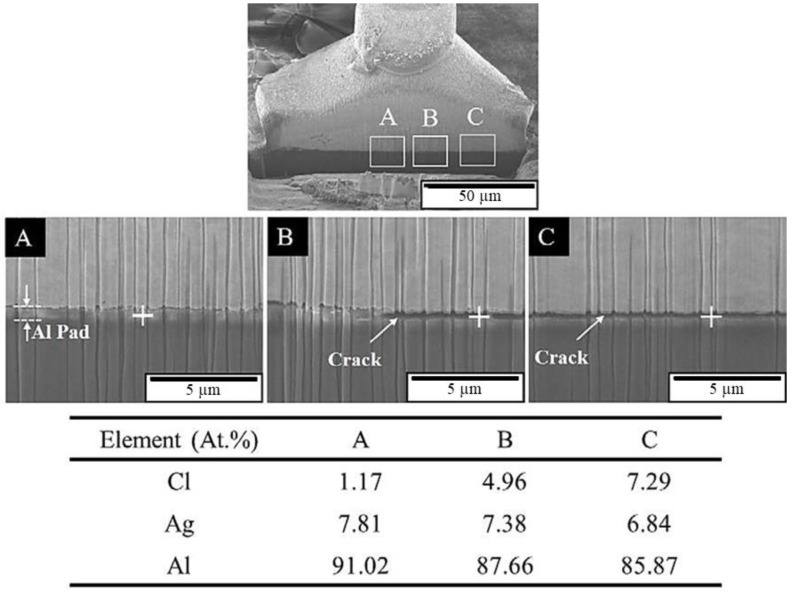
FIB cross-section and EDS element analysis results of the first bond of the APAP wire after 30 min of chlorination.

**Figure 10:**
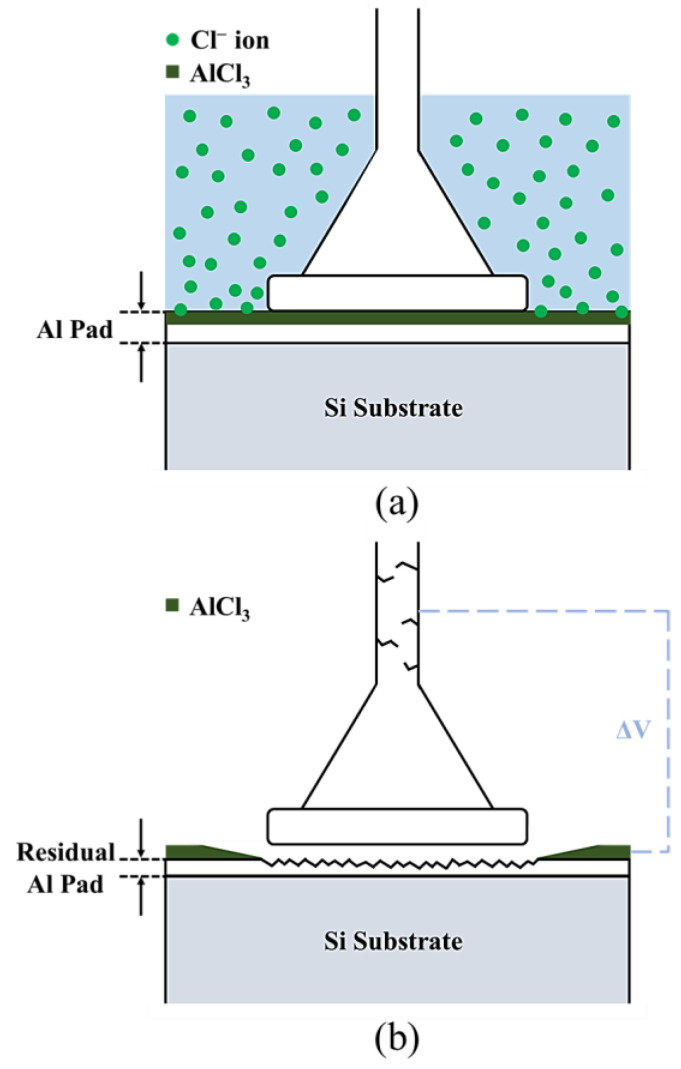
Schematic of the joint of the first bond after chlorination: (**a**) chloride ions corrode the aluminum film; (**b**) the joint of the first bond peels off.

The FIB cross-section and EDS element analysis results of the first bond of the APAP wire after chlorination for 30 min are presented in Figure 9. A clear bond boundary can be identified at the center of the bond. At the periphery of the bond (point C), the silver ball and aluminum film have peeled off, reducing the bond strength. The EDS element analysis reveals a very small chlorine signal at the center of the bond (point A) and a larger chlorine signal at the periphery of the bond (point B). This finding indicated that chlorine ions corroded the aluminum film from the outside to the inside of the bond. As the chlorination time increased, the aluminum film at the bond junction was gradually eroded and hollowed out. Finally, the first bond joint was peeled off and failed (Figure 10).

**Figure 11:**
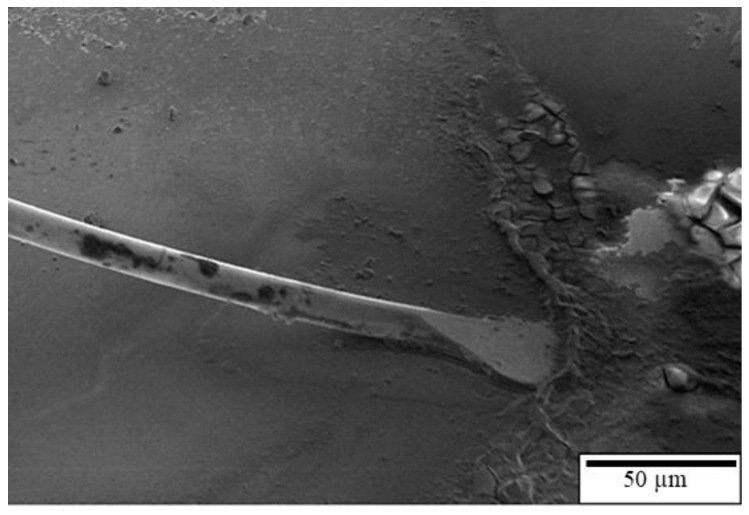
Surface morphology of the second bond of the APAP wire after 30 min of chlorination.

**Figure 12:**
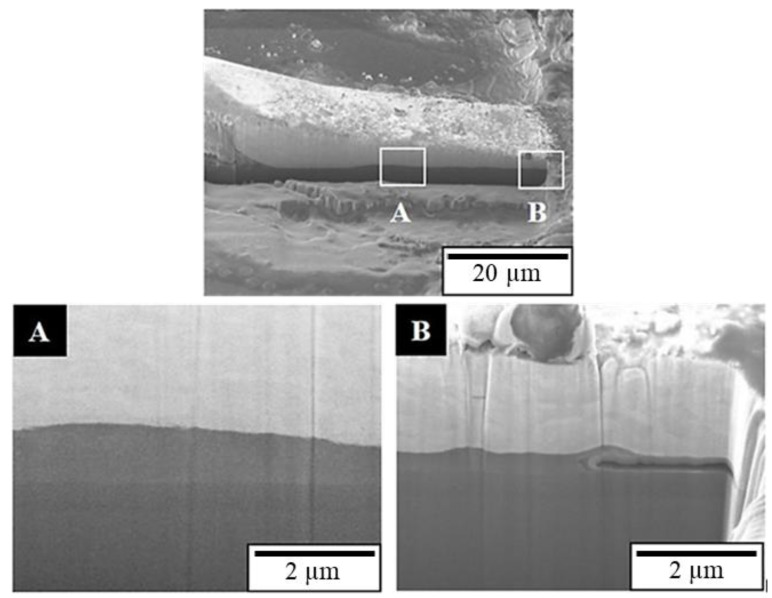
FIB cross-section of the second bond of the APAP wire after 30 min of chlorination. (**A**) Smooth interface; (**B**) aluminum film at the edge is eroded and hollowed out.

**Figure 13:**
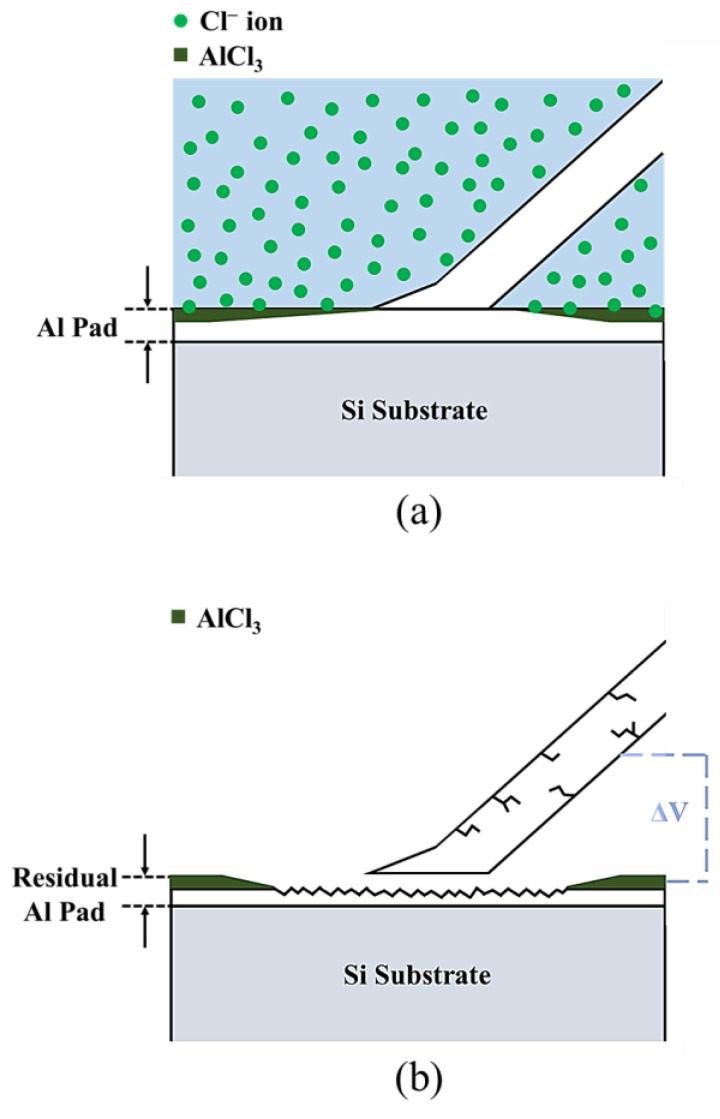
Schematic of the joint of the second bond after chlorination: (**a**) chloride ions corrode the aluminum film; (**b**) partial cracks produced at the joint of the second bond.

Figure 11 presents the surface morphology of the second bond of the APAP wire after 30 min of chlorination, with no bond joint peeling. Figure 12 presents the FIB cross-section analysis results, which reveal that the second bond is well joined; only the aluminum film at the end of the fishtail joint is eroded and hollowed out at approximately 1.5 μm The downforce of the second bond is usually greater than that of the first bond, maintaining a tighter second bond. Therefore, the joint surface of the second bond has greater reliability. A schematic of the chlorination process is presented in Figure 13.

**Figure 14:**
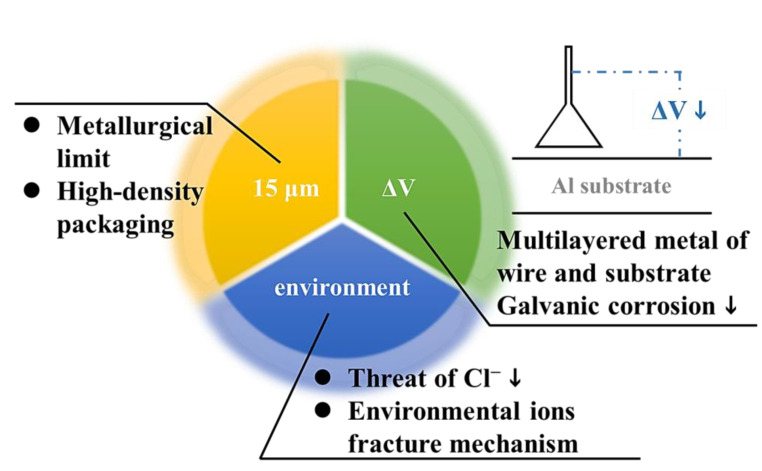
Summary of the study results.

This study has three strengths (Figure 14). First, a quaternary alloy APAP wire was used to replace the common coated wire to reduce the galvanic corrosion caused by the multilayered metal when the wire is bonded to the substrate [28]. Furthermore, the diameter of the quaternary alloy APAP in this study was 15 μm, which is thinner than the silver wire with a diameter of 18–20 μm currently available in the market, reducing the use of precious metals and increasing the packaging density. Finally, we investigated the wire's corrosion fracture in a halogen environment to establish application reference data in depth. The results indicate that the sealant used in the microelectronics packaging industry should contain few chloride ions in the future.

## 4. Conclusions

1. When the APAP wire is immersed in a saturated sodium chloride solution for a long time, chloride ions diffuse into the wire through the grain boundary causing the intergranular fracture to greatly reduce the mechanical properties of the wire;
2. Chloride ions erode the aluminum substrate, causing the aluminum film to become eroded and hollowed out on the joint surfaces of the first and second bonds;
3. With the lower downforce of the first bond, the joint surface is not tight and separates from the Al substrate after 30 min of chlorination. The second bond still combines with the Al substrate at this time.

## Data Availability

The data presented in this study are available on request from the corresponding author. The data are not publicly available due to privacy.

## References

[1] Wu Y.H., Hung F.Y., Lui T.S., Chen K.J. (2019). Study of wire bonding reliability of Ag-Pd-Au alloy wire with flash-gold after chlorination and sulfidation. Microelectron. Reliab..

[2] Chen K.J., Hung F.Y., Chang C.Y. (2019). A Study of the Sulfidation Behavior on Palladium-Coated Copper Wire with a Flash-Gold Layer (PCA) after Wire Bonding. Electronics.

[3] Yang G., Zhou Z., Zhang H., Zhang Y., Peng Z., Gong P., Wang X., Cui C. (2022). Improved Anti-Vulcanization and Bonding Performance of a Silver Alloy Bonding Wire by a Cathodic Passivation Treatment with Palladium. Materials.

[4] Manoharan S., Patel C., McCluskey P. (2017). Advancements in silver wire bonding. International Electronic Packaging Technical Conference and Exhibition, 2017 August, 58097, V001T05A004).

[5] Mazloum-Nejadari A., Khatibi G., Czerny B., Lederer M., Nicolics J., Weiss L. (2017). Reliability of Cu wire bonds in microelectronic packages. Microelectron. Reliab..

[6] Alim M.A., Abdullah M.Z., Aziz M.A., Kamarudin R. (2021). Die attachment, wire bonding, and encapsulation process in LED packaging: A review. Sens. Actuators A Phys..

[7] Mancaleoni A., Sitta A., Colombo A., Villa R., Mirone G., Renna M., Calabretta M. Copper wire bonding process characterization and simulation. Proceedings of the Conference on Integrated Power Electronics Systems (CIPS).

[8] Liu C.P., Chang S.J., Liu Y.F., Su J. (2020). Corrosion-induced degradation and its mechanism study of Cu–Al interface for Cu-wire bonding under HAST conditions. J. Alloys Compd..

[9] Lall P., Deshpande S., Nguyen L. Reliability of copper, gold, silver, and pcc wirebonds subjected to harsh environment. Proceedings of the Electronic Components and Technology Conference (ECTC).

[10] Zhou W.Y., Pei H.Y., Luo J.Q., Wu Y.J., Chen J.L., Kang F.F., Kong J.W., Ji X.Q., Yang G.X., Yang A.H. (2020). Influence of Pd content on microstructure and performance of Au-coated Ag alloy wire. J. Mater. Sci. Mater. Electron..

[11] Ly N., Xu D.E., Song W.H., Mayer M. (2015). More uniform Pd distribution in free-air balls of Pd-coated Cu bonding wire using movable flame-off electrode. Microelectron. Reliab..

[12] Tajedini M., Osmanson A.T., Kim Y.R., Madanipour H., Kim C.U., Glasscock B., Khan M. Electromigration effect on the Pd coated Cu wirebond. Proceedings of the Electronic Components and Technology Conference (ECTC).

[13] Tseng Y.W., Hung F.Y., Lui T.S., Chen M.Y., Hsueh H.W. (2015). Effect of annealing on the microstructure and bonding interface properties of Ag-2Pd alloy wire. Microelectron. Reliab..

[14] Chen C.H., Lee P.I., Chuang T.H. (2022). Microstructure evolution and failure mechanism of electromigration in Ag-alloy bonding wire. J. Alloys Compd..

[15] Tseng Y.W., Hung F.Y., Lui T.S. (2015). Microstructure, mechanical and high-temperature electrical properties of cyanide-free Au-coated Ag wire (ACA). Mater. Trans..

[16] Zhou W., Chen J., Pei H., Kang F., Wu Y., Kong J., Yang G., Yi J. (2019). Microstructure and bonding performance of an Au-coated Ag alloy wire. Microelectron. Eng..

[17] Liqun G., Qiang C., Juanjuan L., Zhengrong C., Jianwei Z., Maohua D., Chung M. (2013). Comparison of Ag Wire and Cu Wire in Memory Package. ECS Trans..

[18] Kai L.J., Hung L.Y., Wu L.W., Chiang M.Y., Jiang D.S., Huang C.M., Wang Y.P. Silver alloy wire bonding. Proceedings of the Electronic Components and Technology Conference (ECTC).

[19] Szymanski R., Charcosset H. (1986). Platinum-Zirconium alloy catalysts supported on carbon or zirconia. Platin. Met. Rev..

[20] Mori K. (2004). Manufacture of Platinum Fibre and Fabric. Platin. Met. Rev..

[21] Huang I.T., Hung F.Y., Lui T.S., Chen L.H., Hsueh H.W. (2011). A study on the tensile fracture mechanism of 15 μm copper wire after EFO process. Microelectron. Reliab..

[22] Tsai H.H., Lee J.D., Tsai C.H., Wang H.C., Chang C.C., Chuang T.H. An innovative annealing-twinned Ag-Au-Pd bonding wire for IC and LED packaging. Proceedings of the International Microsystems, Packaging, Assembly and Circuits Technology Conference (IMPACT).

[23] Wu B.D., Hung F.Y. (2021). Effect of electrification and chlorination on the microstructure and electrical properties of fine Al wires. Microelectron. Reliab..

[24] Yamaji Y., Hori M., Ikenosako H., Oshima Y., Suda T., Umeki A., Kandori M., Oida M., Goto H., Katsumata A. IMC study on Cu wirebond failures under high humidity conditions. Proceedings of the Electronics Packaging Technology Conference (EPTC).

[25] Uno T. (2011). Bond reliability under humid environment for coated copper wire and bare copper wire. Microelectron. Reliab..

[26] Tseng Y.W., Hung F.Y., Lui T.S. (2015). Microstructure, tensile and electrical properties of gold-coated silver bonding wire. Microelectron. Reliab..

[27] Hsueh H.W., Hung F.Y., Lui T.S. (2017). A study on electromigration-inducing intergranular fracture of fine silver alloy wires. Appl. Phys. Lett..

[28] Lee C.S., Tran T., Boyne D., Higgins L., Mawer A. Copper versus palladium coated copper wire process and reliability differences. Proceedings of the Electronic Components and Technology Conference (ECTC).

